# Heterologous prime-boost immunization combining parenteral and mucosal routes with different adjuvants mounts long-lived CD4+ T cell responses in lungs

**DOI:** 10.3389/fimmu.2025.1599713

**Published:** 2025-07-01

**Authors:** Ranmali Kavishna, Thorunn Asta Olafsdottir, Siggeir F. Brynjólfsson, Dennis Christensen, Tobias Gustafsson-Hedberg, Peter Andersen, Manuela Terrinoni, Jan Holmgren, Ali M. Harandi

**Affiliations:** ^1^ Department of Microbiology and Immunology, Institute of Biomedicine, Sahlgrenska Academy, University of Gothenburg, Gothenburg, Sweden; ^2^ Department of Infectious Disease Immunology, Statens Serum Institut, Copenhagen, Denmark; ^3^ Vaccine Evaluation Center, BC Children’s Hospital Research Institute, University of British Columbia, Vancouver, BC, Canada

**Keywords:** adjuvants, prime-boost immunization, parenteral route, mucosal route, T cell response

## Abstract

**Introduction:**

Airway mucosa represents the main entry point for several human pathogens, and as such vaccines against respiratory diseases should ideally elicit protective immune responses in the airways. We have previously reported two immunomodulatory adjuvants based on non-toxic derivatives of Cholera toxin (CT), namely mmCT and CTB-CpG with strong ability to mount mucosal immune responses.

**Methods:**

Herein, we aimed to pinpoint the potential of prime-boost immunization approaches using the fusion-protein based *Mycobacterium tuberculosis* subunit vaccine candidate H56 as a model antigen, combined with adjuvants CAF01, mmCT, and CTB-CpG in mice. This included a parenteral H56+CAF01 priming followed by an intranasal boost with H56+CAF01, H56+mmCT, or H56+CTB-CpG, compared with repeated homologous intranasal administrations of H56 with each adjuvant.

**Results:**

We observed that a parenteral prime with H56+CAF01 followed by an intranasal H56+CTB-CpG booster immunization triggered a Th1-skewed immune response. Conversely, combining the parenteral H56+CAF01 prime with an intranasal H56+mmCT boost resulted in a mixed Th1/Th17-skewed immune response. Notably, the latter combination also engendered anamnestic, long-lived T-cell responses in the lungs which homologous intranasal H56+mmCT immunizations failed to induce.

**Discussion:**

These results suggest that an immunization regimen consists of parenteral priming with H56+CAF01 followed by an airway boosting with H56 protein and mucosal adjuvants holds promise in mounting combined systemic and mucosal immune responses to *Mycobacterium tuberculosis*, and as such warrants further exploration. Given the rising interest in mucosal vaccines for respiratory pathogens, these findings offer an important immunological framework for future translational studies.

## 1 Introduction

Tuberculosis (TB) caused by *Mycobacterium tuberculosis* (Mtb) (1) represents a major global concern with 1.7 billion people estimated to be infected with Mtb; of which 5-15% are expected to develop TB during their lifetime (2). The increase of drug-resistant strains of Mtb complicates treatment and exacerbates global disease burden. Currently, the only commercially-available vaccine against TB is the *Mycobacterium bovis* bacille Calmette–Guerin (BCG). While effective at preventing early manifestations of TB in infants and young children, BCG is reportedly protective for only 10–20 years and has a notoriously variable efficacy in adults in well-controlled field trials (0-80%) (3). Hence, TB represents a formidable threat to public health and underscores the urgent need for an efficacious vaccine. In this regard, a few exploratory vaccines have reached advanced clinical trials, including MTBVAC (4–6), VPM1002 (7–9), GamTBvac (10, 11), M72/AS01E (12, 13) and H56:IC31 (14). The fusion protein H56 consisting of 3 Mtb antigens (Ag85B-ESAT6-Rv2660c) was shown to provide protection against Mtb infection in mice and non-human primates (15, 16). H56 adjuvanted with IC31 (a two-component adjuvant system consisting of antimicrobial peptide [KLK] and TLR9 agonist oligodeoxynocleotide [ODN1a]) (17) was shown to be safe and immunogenic in tuberculosis patients (14).

Vaccines aimed for pathogens such as Mtb that enter the body through the respiratory mucosa should ideally elicit immune responses at the site of infection. Cell-mediated immunity is instrumental in the control of Mtb, particularly CD4+ T cells (18); however a peculiarity in TB is the delayed T cell response observed upon early infection (19). Because mouse model studies have correlated protection to the capacity of Mtb-specific CD4+ T cells to migrate to lung tissue (20, 21) and engage infected antigen-presenting cells (22), a key strategic focus in TB vaccine research is to overcome this delay by eliciting early recruitment of T cells into the infected lung for effective control of Mtb (22, 23). In particular, the benefits of inducing a Th1/Th17 orientation of vaccine-induced immune responses in the lung for immunity against Mtb have been well-described (20, 24), and hence an immunization approach that skews towards such responses is desirable for robust protection.

Early empirical studies with mucosal vaccinations have led to the widely-held view that repeated booster shots of high-dose antigen is needed to reach appropriate levels of immune response, as the mucosal immune compartment is physiologically inclined to prevent overactivation of the immune system (25). Recently, exploiting adjuvants in a “parenteral prime/mucosal pull” regimen has emerged as a strategy for eliciting desirable vaccine-induced immunity in the mucosa. Previous studies have shown that a parenteral prime with H56 adjuvanted by liposomal adjuvant CAF01 followed by an intranasal (i.n.) boost with the same induced cellular immunity in the lung parenchyma (26). Another study reported that H56+CAF01 administered parenterally followed by an intrapulmonary boost elicits mucosal H56-specific T cells and IgA antibody responses, evidencing that both arms of immunity could be induced by such a prime-pull strategy (27). Furthermore, it has been reported that a stronger antigen-specific CD4+ T cell response is induced in mice primed with H56+CAF01 and subsequently intranasally boosted with H56 adjuvanted with TLR9 ligand CpG ODN, compared to mice intranasally boosted with H56 adjuvanted with CAF01 (28). This suggests the value in establishing an optimal combination of adjuvants that are administered in the right order.

We have formerly described two mucosal adjuvants consisting of non-toxic derivates of Cholera toxin (CT): multiple-mutated CT (mmCT); a fully resistant derivative to proteolytic cleavage and lacking enterotoxicity (29), and CTB-CpG; CpG chemically conjugated to the nontoxic B subunit of CT (30). We have previously shown that mice immunized intragastrically with formalin-inactivated *Helicobacter pylori* whole cell vaccine adjuvanted with mmCT showed elevated antibody responses, as well as strong T cell responses with IFNγ/IL-17A production (31). In contrast, when *H.pylori* was adjuvanted with CTB-CpG and administered intranasally, both antibody and T cell responses were elevated with a Th1-skewed orientation (32). We recognize that the chosen animal model limits the generalizability of our findings to the broad human population, including males. However, we view this work as an important first step and future studies will be needed to assess potential sex-based differences and enhance translatability to humans.

Herein, we sought to examine the potential of parenteral-push/mucosal-pull immunization regimes using H56 antigen in combination with CAF01, mmCT or CTB-CpG to mount potent immune responses to H56. We evaluated the resulting T and B cell responses when mice were subcutaneously (s.c.) primed with H56+CAF01 twice and subsequently i.n. boosted once with either H56+CAF01, H56+mmCT or H56+CTB-CpG, in comparison to H56 administered in combination with each adjuvant 3 times i.n. We observed that when a H56+CAF01 parenteral prime was combined with an intranasal H56+CTB-CpG boost, a Th1-skewed immune response was elicited. In contrast, when the parenteral immunization was combined with an i.n. H56+mmCT boost, a mixed Th1/Th17-skewed response was induced. Importantly, we observed that the latter combination also induced long-lived CD4 T cell responses in the lungs while repeated i.n. administrations of mmCT did not, indicating that this immunization schedule warrants further exploration as a potential Mtb vaccine.

## 2 Materials and methods

### 2.1 Mice

Six- to eight-week-old female CB6F1/OlaHsd mice (Harlan Laboratories, The Netherlands) were housed in ventilated cages with free access to food and water. This strain is a first-generation hybrid derived from crossing two strains of mice, the BALB/c (which typically exhibits T2-biased immune response) and C57BL/6 (which exhibits T1 biased response). The balanced T1/T2 immunological profile of this strain is particularly relevant for our study’s objectives of evaluating how different adjuvants and immunization routes can skew the immune response. All mice were housed under standardized pathogen-free conditions at the Experimental Biomedicine Animal Facility, University of Gothenburg.

### 2.2 Ethics statement

The use of mice in this study was performed in accordance with the regulations set forth by the Ethical Committee for Animal Experimentation in Gothenburg, Sweden and in accordance with European Community Directive 86/609. All the techniques and procedures were refined to provide for maximum comfort and minimal stress to mice. Experiments performed were approved by the Ethical Committee for Animal Experimentation in Gothenburg, Sweden under license 64/2015.

### 2.3 Immunization scheme

Groups of mice were either s.c. or i.n. immunized three times, at 21-day intervals. Treatment mice were vaccinated with 5µg recombinant H56 (SSI; diluted in 10mM Tris) combined with either 5µg CTB-CpG (produced as previously described (30)), diluted in Phosphate buffered solution [PBS]), or CAF01 (SSI; diluted in 10mM Tris buffer) mixed in a 1:1 ratio with H56 as recommended by manufacturer. Control mice were administered H56 alone. S.c. administrations were given in volumes of 200 µL, with two 100 µL doses on each side of the base of the tail. Intranasal administrations constituted a volume of 12 µL divided into two equal doses to each nostril. Intranasal administrations adjuvanted with CAF01 were given in small volumes repeatedly 30 minutes apart, comprising a total volume of 40 µL. Unless stated otherwise, heterologous Prime-Boost schedules comprised of two subcutaneous administrations of H56+CAF01 as the prime, and a booster dose of H56 adjuvanted with either CAF01, CTB-CpG or 2 µg mmCT (produced as previously described (29)) administered i.n. A homologous prime-boost immunization scheme consisted of repeated i.n. administrations of H56 adjuvanted with either CAF01, CTB-CpG, mmCT or 2 µg CT (List biological laboratories, Inc; diluted in PBS) 21 days apart.

### 2.4 Serum collection

Blood samples were collected by puncturing the *vena saphena* located in the hindleg two weeks after the final immunization. Samples were left to coagulate at room temperature (RT), then centrifuged 10,000 × *g* for 10 min at 4°C. Sera were frozen at −20°C until further analysis.

### 2.5 Isolation of splenocytes and lung cells

Spleens and lungs were harvested from euthanized mice two weeks post final immunization. Lungs were perfused with 0.1% heparin-PBS, then dried with a towel before one lung from each animal was collected for antibody measurement and the other for FACS analysis. For antibody measurements the lungs were chopped and put into Eppendorf tubes. 30 μL of 20% Saponin was added to each Eppendorf tube, it was then vortexed and stored at 4°C overnight. Next, the lung samples were centrifuged 13,000 × *g* for 10 min and the supernatant stored at -20°C until antibodies were measured with an ELISA. For FACS analysis, one lung from each animal was cut into smaller pieces, resuspended in liberase (1:5 dilution of Liberase [Sigma-Aldrich] in 1640 RPMI [Invitrogen]) in a gentleMACS C tube (Miltenyi Biotech) and dissociated using a gentleMACS dissociator (Miltenyi Biotech). Lungs were then digested for 1hr, 37°C in digestion solution comprising of liberase (1:5 dilution) mixed with 0.5% DNase in RPMI medium (supplemented with 5-10% FCS [Fetal Calf Serum]). Organs were forced through a 70µm nylon mesh cell strainer and single-cell suspensions were collected. Red blood cells from the spleen were lysed with addition of ammonium chloride followed by incubation for 5 minutes at 37°C. RPMI (without FCS) was then added and spun at 500g for 5 minutes. The supernatants were then discarded and the cells were washed again with RPMI. The splenocytes were resuspended in complete RPMI (supplemented with HEPES, penicillin-streptomycin, L-Glutamine, sodium pyruvate and non-essential amino acids and 10% FCS).

### 2.6 Enzyme-linked immunosorbent assay

H56-specific IgG antibody levels in the serum samples were evaluated using an ELISA. Flat 96-well MaxiSorp plates (Nunc) were coated with 0.5 µg/ml H56 in carbonate buffer (pH 9.6) and incubated at 4°C overnight. This was then washed thrice with wash buffer (PBS with 0.2% Tween-20), and then blocked with blocking buffer (PBS with 2% Bovine Serum Albumin [BSA]) for 2 hours at room temperature. The plates were then washed another three times, followed by incubation with serially diluted serum samples (diluted in PBS with 1% BSA), starting with a dilution of 1:3 for IgA evaluation and 1:10 for IgG evaluation. Background wells without serum samples were included as controls. After incubation, plates were washed another three times with washing buffer. Dilutions of HRP-conjugated secondary anti-mouse antibodies (Invitrogen) were made in PBS with 1% BSA as follows: 1:20000 for anti-mouse IgG antibody, and 1:5000 for anti-mouse IgA antibody. Secondary antibodies were then added to wells and incubated for 1hr at room temperature. This was then followed by washing five times with washing buffer. For the last wash, wash buffer was left in wells for 1 minute before discarding. TMB Ready-to-use substrate (Kim-En-Tec) was then added to the wells. Reaction was stopped after 10 minutes with stopping solution (H_2_SO_4_ 2N). Absorbance was then read at 450nm. Endpoint titers were assessed by plotting curves as Log_10_ values on GraphPad PRISM and fitting sigmoidal curves without constraint.

### 2.7 Antigen-recall stimulation and intracellular cytokine assessment

Lung cells and splenocytes were harvested from euthanized mice as described above. Cell suspensions were resuspended in complete RPMI (supplemented with HEPES, penicillin-streptomycin, L-Glutamine, sodium pyruvate, non-essential amino acids and 10% FCS), adjusted to 1 x 10^7^ cells/mL and were seeded in v-bottom 96-well plates. For antigen recall stimulation, 2 µg/ml of H56 was added to each well. A negative medium-only control and a positive control of PMA (Sigma-Aldrich; 50 ng/mL) + ionomycin (Sigma-Aldrich; 1µM) were included for each sample. Single stain-only controls were also included for compensation purposes, as well as an unstained control. Anti-CD28 (BD Biosciences) and anti-CD49d (BD Biosciences) antibodies were added to a final concentration of 1 µg/mL. The plates were then incubated for 2 hours at 37°C. Brefeldin A (Sigma-Aldrich) was then added to the wells to a final concentration of 10 µg/ml and incubated for 5 hours at 37°C using ThermostatPlus heatblock (Eppendorf) programmed to cool down to 4°C for the next day. The plates were then spun at 2200 rpm for 3 mins at 4°C. The supernatants were then discarded, and the cells resuspended in FACS buffer (PBS with 1% FCS). The cells were spun at 2200 rpm for 3 minutes and supernatants discarded. They were then blocked with anti-CD16/32 antibody (BD Pharmingen) for 10 min at room temperature. Cells were then surface stained with CD4:PE-CF594 (eBioscience), CD44:V450 (BD Biosciences) and CCR6:AF647 (eBioscience) (all dilutions were 1:200) and incubated for 15 minutes in the dark. Cells were then spun at 2200 rpm for 3 mins at 4°C, supernatant discarded, and washed again in FACS buffer as before. For intracellular staining, cells were fixed with the addition of Cytofix/Cytoperm (BD Biosciences) and incubated for 20 minutes at 4°C in the dark. Perm Wash was then added to wells, and plates spun at 2200 rpm for 3 minutes and supernatant discarded. Intracellular antibodies were then diluted 1:200 in Perm Wash Buffer: IFNγ:FITC (eBioscience), TNFα:PE-Cy7 (eBioscience) and IL-17:PerCP-Cy5.5 (eBioscience) and added. Plates were incubated for 20 minutes at 4°C in the dark. Cells were washed thrice in Perm Wash, resuspended in FACS buffer and then subjected to flow cytometry on LSR II (BD Biosciences). Cytokine-secreting CD4+ T cells were then evaluated by assessing IFN-γ, TNF-α & IL-17 positive gates within the CD4+ CD44^High^ cell population. Boolean combination gates were used to assess the frequency of each cytokine co-expressing subset and its frequency in total T cell population.

### 2.8 Th1/Th2/Th17-specific cytokine evaluation using bead array

Th1/Th2/Th17-specific cytokines were measured in H56-induced proliferated T cells using bead array. The BD™ CBA mouse Th1/Th2/Th17 Cytokine Kit (BD Biosciences) was used as per manufacturer’s instructions to evaluate levels of IL-2, IL-4, IL-6, IFN-γ, TNF-α, IL-17A, and IL-10. Briefly, capture beads for each cytokine were combined and mixed with dilutions of splenocytes or recombinant standard. These were then incubated with PE-conjugated detection antibodies to form sandwich complexes. The intensity of PE fluorescence of each sandwich complex correlates with the concentration of cytokine. Samples were then run on LSR II (BD Biosciences), and FCAP Array™ software was utilized to generate results in graphical and tabular format.

### 2.9 Statistics

Data analyses were performed using Graphpad Prism 7.0. Statistical comparison was done using non-parametric Mann-Whitney test, Kruskal-Wallis test, or an ordinary one-way ANOVA. Differences were considered significant (*) at *p* value <0.05.

## 3 Results

### 3.1 Differential immune profiles induced by CAF01 and CTB-CpG when administered together with H56 parenterally or mucosally

**Figure 1:**
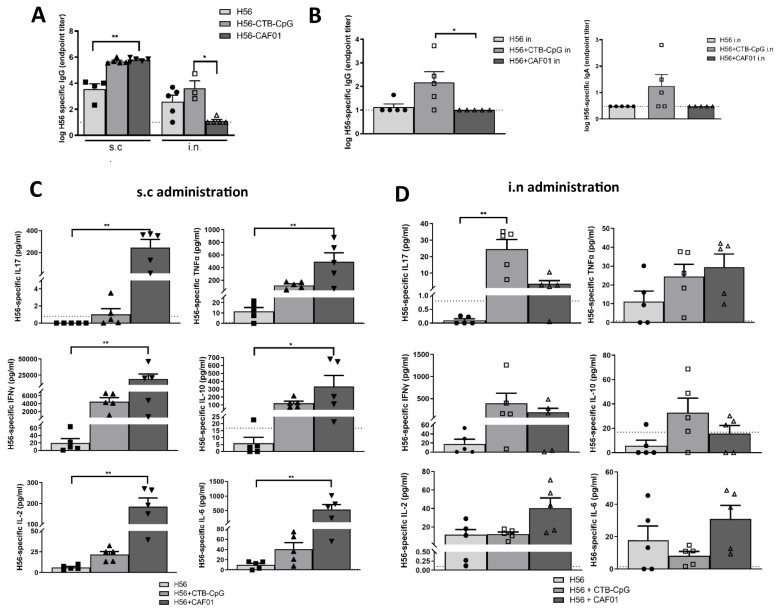
Immune signatures induced upon parenteral and instranasal administrations of CTB- CpG and CAF01. Mice were immunized with either H56-CTB-CpG or H56-CAF01 either s.c or i.n (n=3 to 5). H56-specific antibody levels were measured via ELISA in the serum **(A)** and lungs **(B)**. The dashed line represents the lowest dilution of the sera, and a nominal titer of either 10 (IgA) or 3 (IgG) was assigned to samples with a titer below the lowest dilution. Log-transformed H56-specific titers were compared, and statistical significance was assigned to the differences. Splenocytes were harvested post-vaccination and restimulated with H56 to measure vaccine-induced cellular response. Cytokines were measured via bead array in s.c immunized mice **(C)** or i.n immunized mice **(D)**. The dashed line shows lower limit of quantification. Error bars show mean + SEM. Data were analyzed using a Kruskal-Wallis test and differences were considered statistically significant at *p* values of <0.05 (*), <0.01 (**).

We first immunized mice either s.c. or i.n. with H56 adjuvanted with either CAF01 or CTB-CpG to evaluate their systemic humoral immune response. We observed that while s.c. administration of both H56+CAF01 and H56+CTB-CpG resulted in strong serum H56-specific IgG antibody levels, i.n. administration of H56+CAF01 resulted in a poor vaccine-specific humoral response compared to H56+CTB-CpG (**Figure 1A**). Further, i.n. administration of H56+CTB-CpG enhanced both vaccine-specific IgG and IgA antibody levels in the lungs, which was not observed with i.n. administration of H56+CAF01 (**Figure 1B**).

We then isolated splenocytes of vaccinated mice and restimulated with H56 to evaluate T cell-associated cytokine response. Interestingly, splenocytes from mice immunized s.c. with H56+CAF01 generated stronger IL-17, TNF-α, IFN-γ, IL-1+, IL-6 and IL-2 cytokine levels than that from s.c. H56+CTB-CpG-immunized mice (**Figure 1C**). This suggests that while parenteral administration of both vaccines results in a similar systemic humoral response, H56+CAF01 may induce a stronger peripheral T cell response. In contrast, i.n. administration of the two vaccines resulted in distinct cytokine profiles. While splenocytes from H56+CAF01-immunized mice had higher levels of IL-6 and IL-2 levels than H56+CTB-CpG-immunized mice, the latter showed stronger IL-17 (p<0.01), IFN-γ and IL-10 levels (suggestive of a Th17-skewed immune response) (**Figure 1D**).

These results collectively point to the suitability of CTB-CpG as a mucosal adjuvant because its i.n administration elicited systemic T cell immune responses, as well as humoral responses in both the lungs and periphery. In contrast, while s.c. administered CAF01 induced robust systemic cellular and humoral responses, poor mucosal responses were induced upon i.n. administration.

### 3.2 Heterologous prime-boost strategies elicits potent mucosal humoral immunity

**Figure 2:**
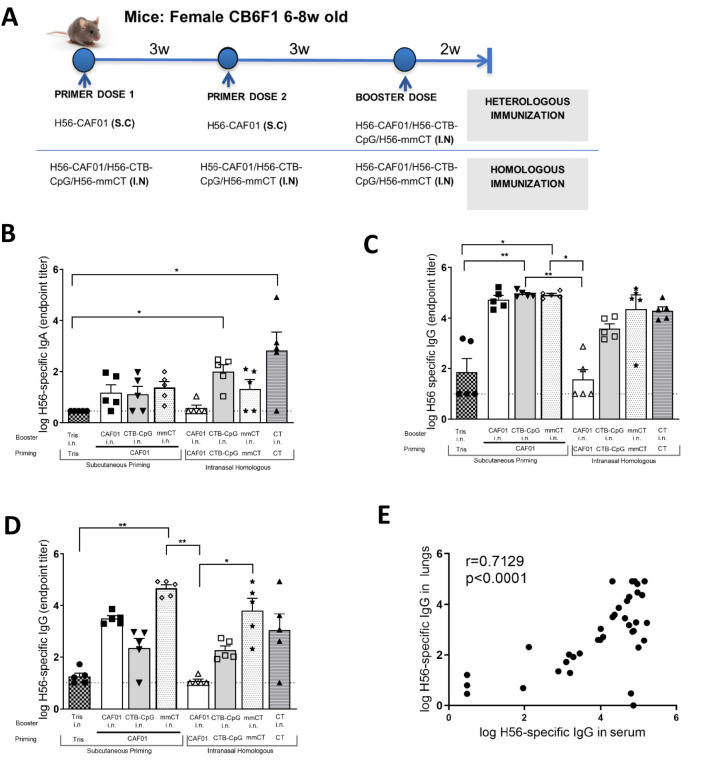
Humoral responses in serum and lungs 2 weeks after booster dose. Mice were either heterologously or homologously immunized with H56 and different adjuvants as indicated (n=5). Control mice were given Tris-H56 at each immunization. A group administered homologous H56 together with Cholera-toxin (CT) was included as a positive control for i.n. immunization **(A)**. Antibody response was evaluated as described in the legend of **Figure 1**, 2 weeks after last booster dose. H56-specific IgA levels in the lungs **(B)**, and IgG levels in the serum **(C)** and lungs **(D)** were measured. Error bars show mean + SEM. Pearson correlation coefficient (r) was calculated between vaccine-specific lung IgG levels and serum IgG levels **(E)**. Data in **(B-D)** were analyzed using a Kruskal-Wallis test followed by a Dunn’s multiple comparisons test between all groups. Differences were considered statistically significant at *p* values of <0.05 (*), <0.01 (**).

We sought to determine whether a heterologous prime-boost immunization scheme consisting of a strong parenteral adjuvant (CAF01) as the prime, and strong mucosal adjuvants (CTB-CpG and mmCT) as i.n. boosters would enhance the mucosal immune response. We compared this to repeated homologous i.n. administrations of the same adjuvant, and also included CT as a gold standard adjuvant for homologous mucosal immunization. Mice were immunized either homologously with repeated i.n. administrations of the same adjuvant, or heterologously with a parenteral prime (H56+CAF01) and a mucosal boost of either H56+CAF01, H56+CTB-CpG or H56+mmCT (**Figure 2A**). To evaluate the humoral response induced by the different prime-boost regimens, we measured IgG and IgA antibody levels in serum and perfused lungs. We observed that the H56-specific IgA antibody response induced in the lungs were highest in the group that received repeated intranasal CT immunizations. However, IgA levels across all 3 heterologous prime-boost immunization groups were comparable (**Figure 2B**). Compared to the homologous i.n. groups, with the exception of the combination of H56+CAF01 s.c./H56+CAF01 i.n., heterologous prime-boost scheme did not provide an added benefit in inducing vaccine-specific IgA antibody levels in the lungs (**Figure 2B**).

This trend was similarly observed in the induction of serum and lung H56-specific IgG antibody levels. While heterologous vaccine regimes induced H56-specific IgG antibody levels in both sera (**Figure 2C**) and lungs (**Figure 2D**), it was not superior to that seen by their homologous intranasal counterparts – with the exception of H56+CAF01 s.c./H56+CAF01 i.n. in the lungs. Furthermore, the IgG levels in the lungs strongly correlated (r=0.71, p<0.0001) with IgG antibody levels in the serum (**Figure 2E**) suggesting transudation of serum IgG antibody from the blood circulation in to the lung mucosa, a phenomenon that has been reported previously (33). Taken together, these results demonstrate that heterologous prime-boost may be beneficial to evoke a balanced systemic and mucosal responses that are non-inferior to that observed with homologous i.n. prime-boost combinations with strong mucosal adjuvants.

### 3.3 The choice of mucosal adjuvant used for the booster response shapes the Th cell response recruited to the lungs

**Figure 3:**
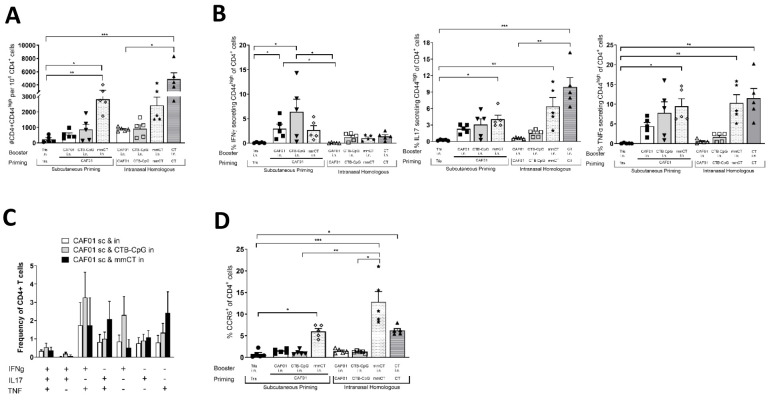
Mucosal CD4+ T cell response post-homologous and heterologous immunization. Mice were immunized as previously described in **Figure 2** legend (n=5). Lung cells were isolated 2 weeks post-booster immunization and restimulated with H56 antigen *in vitro.* Cells were stained with fluorescently labeled antibodies and analyzed with flow cytometry to evaluate total CD4+ T cell count **(A)**, frequency of cytokine-secreting CD4+ T cells **(B)**, frequency of double and triple cytokine-secreting CD4+ T cells **(C)**, and frequency of CCR6+ CD4+ T cells **(D)**. Error bars show mean + SEM. Data were analyzed using a Kruskal-Wallis test followed by a Dunn’s multiple comparisons test. Differences from the control were considered statistically significant at *p* values of <0.05 (*), <0.01 (**), and <0.001 (***).

Next, we sought to investigate whether the different prime-boost strategies would differentially skew the Th profile in the lung mucosa. To this end, CD4+ T cells were retrieved from mice immunized with different prime-boost regimens two weeks after the last immunization and performed CD4+ T cell-specific and intracellular cytokine analysis upon H56 restimulation. The group that received homologous CT i.n. administrations had the highest frequency of antigen-specific CD4+ T cells (CD44^high^) in lungs compared to all other groups (**Figure 3A**). We observed that i.n CT immunization exhibited a strong Th17 profile, as indicated by the secretion of IL-17 and TNFα (**Figure 3B**).

Within the heterologous prime-boost groups, the number of antigen-experienced CD4+ T in lungs of the groups receiving H56+CAF01 s.c./H56-mmCT i.n were higher than in the other heterologous prime-boost groups (**Figure 3A**). In addition, the prime-boost combination of H56+CAF01 s.c./H56+CTB-CpG i.n. resulted in a stronger Th1-skewed profile than the other heterologous groups, as evidenced by stronger IFN-γ-secreting CD4+ T cells (**Figure 3B**). This is further supported by higher levels of IFN-γ+TNFα double-secreting or IFN-γ single-secreting cells in H56+CAF01 s.c./H56+CTB-CpG i.n. than the other heterologous immunization groups (**Figure 3C**). In contrast, a combination of H56+CAF01 s.c./H56+mmCT i.n. resulted in a polyfunctional Th1/Th17 immune phenotype, as evidenced by strong levels of IL-17+TNFα double secreting cells (**Figure 3C**) and higher levels of CCR6+ CD4+ T cells (p<0.05, **Figure 3D**). Further, cytokines detected in the cell culture supernatant from H56-restimulated lung-derived CD4+ T cells also supported that heterologous H56+CAF01 s.c./H56+CTB-CpG i.n. mainly induced a Th1 response whereas H56+CAF01 s.c./H56+mmCT i.n. was more Th17 biased (**Supplementary Figure 1**). We also observed that while the heterologous H56+CAF01 s.c./H56+mmCT i.n group engendered similar levels of H56-responding CD4+ T cells as those received homologous H56-mmCT intranasal administrations (**Figure 3A**), the latter combination induced a higher frequency of H56-specific CCR6+ CD4+ T cells (**Figure 3D**).

These results suggest that by varying the mucosal booster, a uniquely Th1-skewed or Th1/Th17-skewed mucosal response can be induced. Importantly, replacing the parental prime with either H56+CTB-CpG or H56+mmCT (followed by i.n. immunization with the same adjuvant) induces limited mucosal T-cell responses (**Supplementary Figure 2**). These results suggest that the enhanced mucosal immunity induced by the parenteral administration of CAF01 followed by a mucosal administration, was unique to CAF01. Moreover, this effect is not seen when a parenteral CAF01 administration is preceded by intranasal CTB-CpG (**Supplementary Figure 3**), indicating that the order of administration is important.

### 3.4 Heterologous CAF01 prime/mmCT boost results in mucosally directed long-lived CD4+ T cell and B cell responses

**Figure 4:**
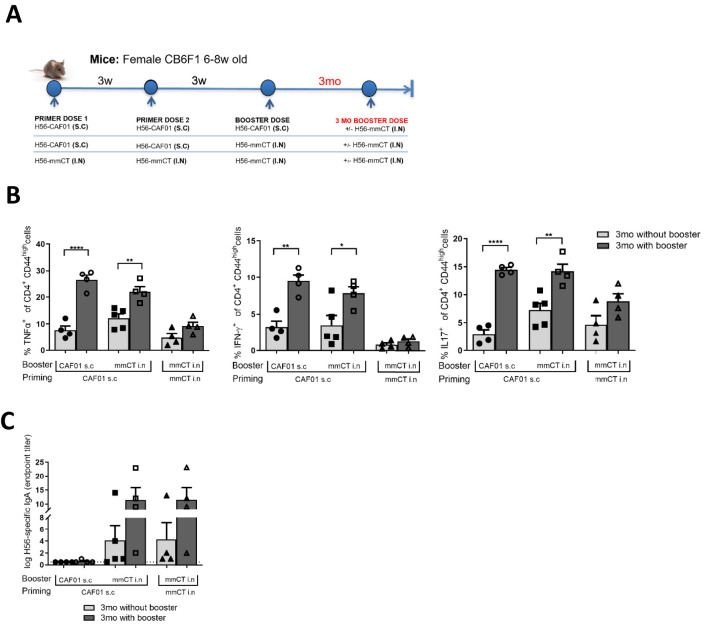
Long-lived mucosal T cell responses following immunization. Mice were either homologously s.c. immunized with CAF01 or i.n. with mmCT, or heterologously with s.c. CAF01 and boosted with i.n mmCT, with or without a booster dose of mmCT at 3months (n=4 to 5) **(A)**. Lung cells were isolated 2 weeks post-final booster immunization and restimulated with H56 antigen *in vitro*. Cells were stained with fluorescenty labeled antibodies and analyzed with flow cytometry to evaluate frequency of cytokine-secreting CD4+ T cells **(B)**. Additionally, H56-specific IgA titers were evaluated by ELISA as described in the legend of **Figure 1** **(C)**. Error bars show mean + SEM. Data were analyzed using an ordinary one-way ANOVA followed by a Tukey’s multiple comparisons test with a single pooled variance. Differences were considered statistically significant at *p* values of <0.05 (*), <0.01 (**) and <0.0001 (****).

Next, we examined the longevity of the mucosal immune responses induced by the heterologous parenteral prime/mucosal boost immunization. Thus, we carried out a 3-month long study with the following groups: homologous parenteral immunization (H56+CAF01 s.c.), heterologous prime-boost (H56+CAF01 s.c. followed by H56+mmCT i.n.) and homologous mucosal immunization (repeated H56+mmCT i.n.). Three months after the last immunization, half of the mice received an additional i.n. booster with mmCT for evaluation of mucosal recall responses (**Figure 4A**). Firstly, we observed detectable levels of H56-specific cytokine-secreting CD4^+^ T cells in the lungs of all immunized groups 3 months following the last immunization (**Figure 4B**). These levels were further increased after the 3-month booster in the groups that had been parenterally primed with CAF01, whereas no significant booster effects were observed in the groups that had only received repeated i.n. immunization (**Figure 4B**). Further, the frequency of IFN-γ-secreting and TNFα-secreting CD4+ T cells post-boost in mice that received H56+CAF01 s.c./H56+mmCT i.n. immunization was higher than that observed in mice with repeated i.n. mmCT immunizations. This indicates that while we had previously observed no added benefit of a CAF01 s.c/mmCT i.n combination compared to repeated mucosal mmCT vaccinations in terms of humoral (**Figure 2**) and cellular immune responses in the mucosa (**Figure 3**) 2 weeks post-final immunization, CAF01 s.c/mmCT i.n immunization did result in a more profound T cell anamnestic response 3 months post-final immunization. Moreover, while homologous s.c. administration of H56+CAF01 showed a comparable trend to that of heterologously administered H56+CAF01s.c/H56+mmCT i.n in terms of mucosal T cell response, the former elicited undetectable mucosal IgA antibody levels, even upon the 3-month booster (**Figure 4C**). In contrast, the heterologous immunization regimen induced enhanced mucosal IgA antibody levels indicating the induction of a strong mucosal memory B cell response; however these levels were not different from that observed in the group that received repeated i.n. administrations (**Figure 4C**). Collectively, these results indicate that the heterologous prime-boost immunization with H56+CAF01 s.c./H56-mmCT i.n. is beneficial to induce potent long-lived H56-specific CD4+ T cell and IgA responses that can be pulled to the mucosal tissue upon i.n. booster.

## 4 Discussion

Vaccines that are efficacious in preventing TB in adults and adolescents are in urgent need to reduce the global disease burden of TB, and the induction of immunity in the airway mucosa is highly desirable in effective Mtb control. Herein, we have characterized the systemic and mucosal immune responses induced following immunization with H56+CAF01, H56+mmCT and H56+CTB-CpG in various homologous and heterologous immunization schemes.

We observed that parenteral administration of H56+CTB-CpG or H56+CAF01 induced a strong systemic H56-specific antibody and T cell responses. Moreover, H56+CAF01 administered s.c. induced a more profound systemic T cell response than H56+CTB-CpG. Intranasal administrations of both vaccines also induced vaccine-specific cellular responses in the periphery, however only H56+CTB-CpG induced H56-specific humoral response in both the lung or serum. Intranasally administered H56+CAF01 was unable to mount any appreciable level of H56-specific mucosal antibodies in either the periphery or lung. Repeated intranasal administrations of CAF01 in a homologous regime (used as both prime and boost) also resulted in weak mucosal immune responses. This indicates that CAF01 is not by itself an optimal nasal adjuvant for the induction of humoral and cell-mediated responses. Nevertheless, our results showed that the immunomodulatory properties of a parenterally-administered CAF01 can be leveraged in terms of mucosal immunity if combined with an i.n. booster using a potent mucosal adjuvant. In this regard, i.n. boosting of heterologously immunized mice with CAF01, mmCT and CTB-CpG elicited stronger mucosal responses than repeated homologous i.n.-administeration of CAF01.

Several studies have documented that CD4+ T cells play an essential role in mediating protection against TB in both humans and in animal models (34, 35). IFN-γ produced by Th1 cells facilitates bacterial clearance by stimulating phagocytic activity and reactive oxygen species secretion in macrophages (36), but there is mounting evidence that Th1-immunity alone is insufficient for protection. Indeed, the MV85A vaccine trial serves as a cautionary tale where the generation of a strong IFN-γ-secreting CD4+ T cell response failed to translate to additional protection against TB (37). It is becoming increasingly evident that in addition to Th1 responses, an IL-17+/Th17 response is crucial for protection against Mtb (38, 39), with studies showing that IL-17 is a key mediator of protection against Mtb (40, 41), and the capacity of Th17 cells to transform into lung resident lymphocytes has been well-documented (42). Without being able to directly test the efficacy of the different prime-boost vaccination in a challenge model, we focused on characterizing the CD4^+^ T cell responses as correlates of protection. Our results herein showed that mice given H56+CAF01 s.c/H56+CTB-CpG i.n exhibited an IFN-γ-skewed T cell immune response in the lungs, while those given H56+CAF01 s.c/H56+mmCT i.n showed a mixed cytokine response secreted by CCR6-expressing T cells, dominated by IL-17 and TNFα double-secreting cells.

Interestingly, we observed that the heterologous immunizations with H56+CAF01 s.c/H56+CTB-CpG i.n and H56+CAF01 s.c./H56+mmCT i.n did not provide an added benefit to homologous i.n immunization with H56+CTB-CpG or H56+mmCT in terms of mucosal H56-specific antibodies and cell-mediated cytokine responses. However, assessing the long-lived recall response at 3 months revealed a crucial difference. Despite the strong mucosal Th17 response induced following homologous i.n H56+mmCT/H56+mmCT, this immunization scheme failed to mount a recall T-cell response upon a booster immunization 3 months later. This contrasted with both the heterologous H56+CAF01 s.c/H56+mmCT i.n group and the homologous parenteral H56+CAF01 s.c./H56+CAF01 s.c. group, which exhibited a strong anamnestic TNFα/IFN-γ/IL-17-secreting CD4+ T cell response in the lungs. A more in-depth investigation will be needed to verify the phenotype of the memory T cell subsets in the airway mucosal compartment.

The mechanics of how a heterologous vaccination strategy results in a significant induction of long-lived effector memory CD4+ T cells in the lungs upon the recall booster shot at 3 months warrants further investigation. Previous studies have observed the phenomenon of memory cells induced by systemic priming that are then recruited to the mucosal site upon recall – thereby enhancing the immunogenicity of a mucosal vaccine (43, 44).

The study presented herein however is limited by the lack of an Mtb challenge study to directly assess if and how long-lived T cell immunity elicited in the lungs of heterologously immunized mice can translate to protection. Furthermore, more detailed investigation will be needed to elucidate the contribution of local antibody secretion in the lungs versus transudation from the circulation. Collectively, this study has demonstrated the value of a rational vaccine design where combining parenteral and mucosal routes together with selection of the adjuvants used can both direct the type of immune response required and potentially affect the longevity of the immune response.

## Data Availability

The raw data supporting the conclusions of this article will be made available by the authors, without undue reservation.

## Supplementary material

Supplementary Figure 1 Th1/Th2/Th17-specific cytokines in lungs post-immunization. Mice were immunized as described in **Figure 2** (n=2 to 5). Lungs were harvested 2 weeks post-booster and cells incubated with capture beads to evaluate cytokines. Dashed line shows lower limit of quantification. Error bars show mean + SEM. Data were analyzed using a Kruskal-Wallis test followed by a Dunn’s multiple comparisons test. Differences from the control were considered statistically significant at *p* values of <0.05 (*), <0.01 (**), and <0.001 (***).

Supplementary Figure 2 Mucosal CD4+ T cell response post-subcutaneous prime and intranasal boost. Mice were subcutaneously primed with either H56-CTB-CpG or H56-mmCT and intranasally boosted with the same adjuvant as indicated (n=5) **(A)**. Lung cells were isolated 2 weeks post-booster and restimulated with H56 antigen *in vitro*. Cells were stained with fluorescently labeled antibodies and analyzed with flow cytometry to evaluate frequency of cytokine-secreting CD4+ T cells **(B)**. Error bars show mean + SEM. Data were analyzed using a Mann-Whitney test. Differences were considered statistically significant at *p* values of <0.05 (*) and <0.01 (**).

Supplementary Figure 3 Mucosal CD4+ T cell response post-intranasal prime and subcutaneous boost. Mice were intranasally primed with either H56-CTB-CpG or H56-mmCT, and subcutaneously boosted with the same adjuvant as indicated (n=5) **(A)**. Lung cells were isolated 2 weeks post-booster and restimulated with H56 antigen *in vitro*. Cells were stained with fluorescenty labeled antibodies and analyzed with flow cytometry to evaluate frequency of cytokine-secreting CD4+ T cells. Subcutaneously primed groups from **Figure 3** (CAF01 s.c./CTB-CpG i.n and CAF02 s.c./mmCT i.n.) are included for comparison **(B)**. Error bars show mean + SEM. Data were analyzed using a Kruskal-Wallis test followed by a Dunn’s multiple comparisons test. Differences from the control were considered statistically significant at *p* values of <0.05 (*).

Supplementary Figure 4 Gating strategy for mucosal CD4+ T cell cytokine response post-vaccination. Mice were either vaccinated as described in **Figure 3**, **Figure 4**, **Supplementary Figure 2** or **Supplementary Figure 3** legends. Lung cells were isolated two weeks after the booster dose and restimulated *in vitro* with H56 antigen or left unstimulated (negative control). PMA stimulation was included as positive control. Cells were stained with fluorescently labeled antibodies and analyzed by flow cytometry to assess cytokine production by CD4^+^CD44^+^ T cells. Representative gating strategy employed **(A)**, and control plots from a single mouse used to define cytokine-positive gates based on unstimulated (negative) and PMA-stimulated (positive) conditions **(B)** are shown.
