# Supplementary material for: Heterologous prime-boost immunization combining parenteral and mucosal routes with different adjuvants mounts long-lived CD4+ T cell responses in lungs
Source: Front Immunol. 2025 Jul 1;16:1599713. doi: 10.3389/fimmu.2025.1599713 (PMC12259646; doi:10.3389/fimmu.2025.1599713)
Supplement: Supplementary Figure 1 — Th1/Th2/Th17-specific cytokines in lungs post-immunization. Mice were immunized as described in Figure 2 (n=2 to 5). Lungs were harvested 2 weeks post-booster and cells incubated with capture beads to evaluate cytokines. Dashed line shows lower limit of quantification. Error bars show mean + SEM. Data were analyzed using a Kruskal-Wallis test followed by a Dunn’s multiple comparisons test. Differences from the control were considered statistically significant at p values of <0.05 (*), <0.01 (**), and <0.001 (***). [file DataSheet1.pdf]

SUPPLEMENTARY FIGURES

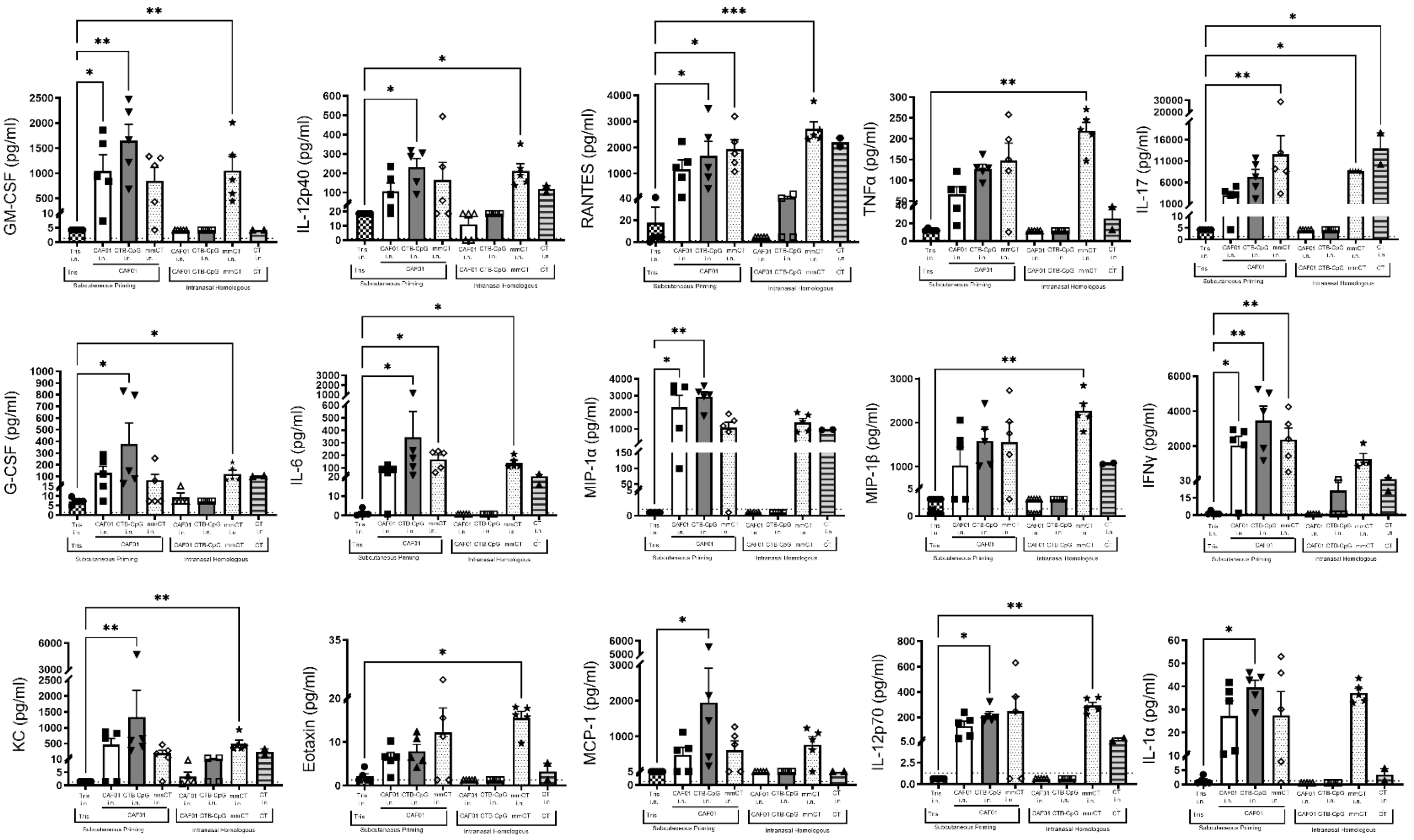

Supplementary Figure 1: Th1/Th2/Th17-specific cytokines in lungs post-immunization. Mice were immunized as described in Figure 2 (n=2 to 5). Lungs were harvested 2 weeks post-booster and cells incubated with capture beads to evaluate cytokines. Dashed line shows lower limit of quantification. Error bars show mean + SEM. Data were analyzed using a Kruskal-Wallis test followed by a Dunn's multiple comparisons test. Differences from the control were considered statistically significant at *p* values of <0.05 (*), <0.01 (**), and <0.001 (***)

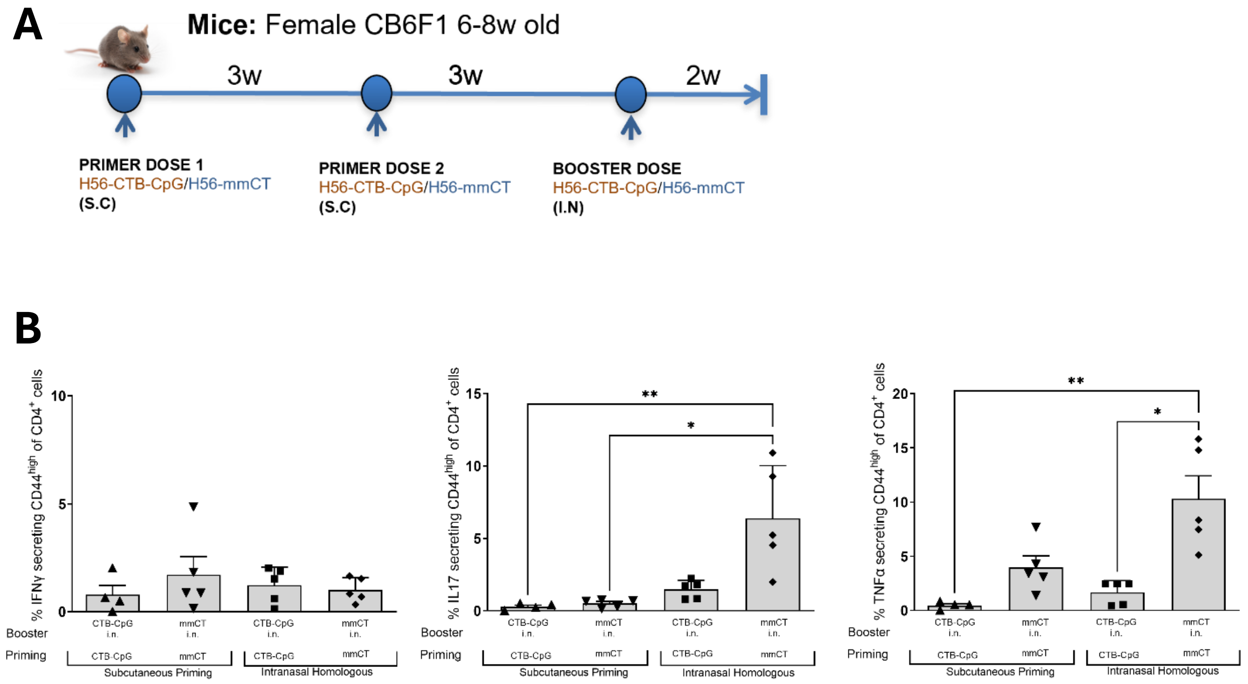

Supplementary Figure 2: Mucosal CD4+ T cell response post-subcutaneous prime and intranasal boost. Mice were subcutaneously primed with either H56-CTB-CpG or H56-mmCT and intranasally boosted with the same adjuvant as indicated (n=5) (A). Lung cells were isolated 2 weeks post-booster and restimulated with H56 antigen *in vitro*. Cells were stained with fluorescently labeled antibodies and analyzed with flow cytometry to evaluate frequency of cytokine-secreting CD4+ T cells (B). Error bars show mean + SEM. Data were analyzed using a Mann-Whitney test. Differences were considered statistically significant at *p* values of <0.05 (*) and <0.01 (**)

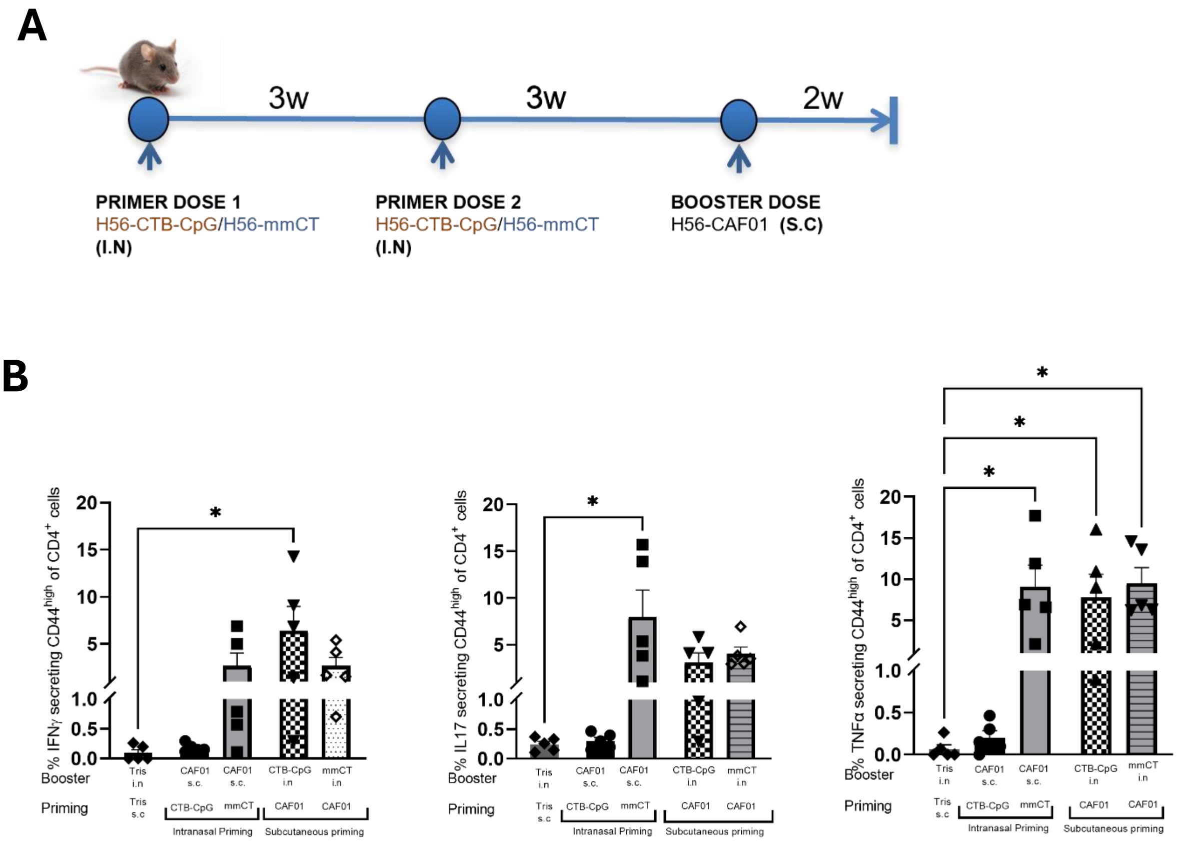

Supplementary Figure 3: Mucosal CD4+ T cell response post-intranasal prime and subcutaneous boost. Mice were intranasally primed with either H56-CTB-CpG or H56-mmCT, and subcutaneously boosted with the same adjuvant as indicated (n=5) (A). Lung cells were isolated 2 weeks post-booster and restimulated with H56 antigen *in vitro*. Cells were stained with fluorescenty labeled antibodies and analyzed with flow cytometry to evaluate frequency of cytokine-secreting CD4+ T cells. Subcutaneously primed groups from Figure 3 (CAF01 s.c./CTB-CpG i.n and CAF02 s.c./mmCT i.n.) are included for comparison (B). Error bars show mean + SEM. Data were analyzed using a Kruskal-Wallis test followed by a Dunn's multiple comparisons test. Differences from the control were considered statistically significant at p values of <0.05 (*).

A

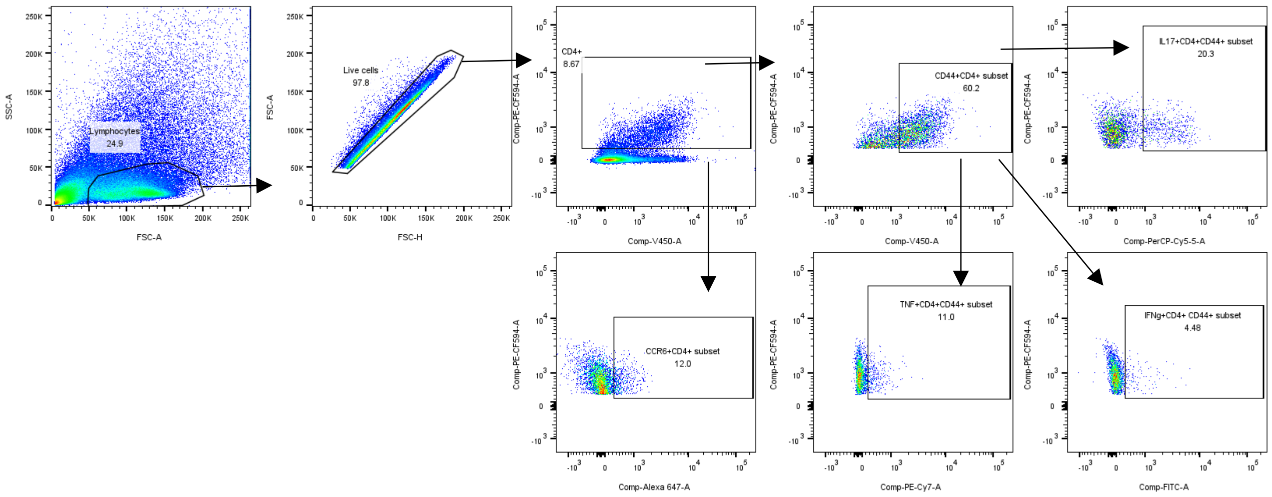

B

Unstimulated (Negative control) **H56-stimulated** **PMA-stimulated (Positive control)**

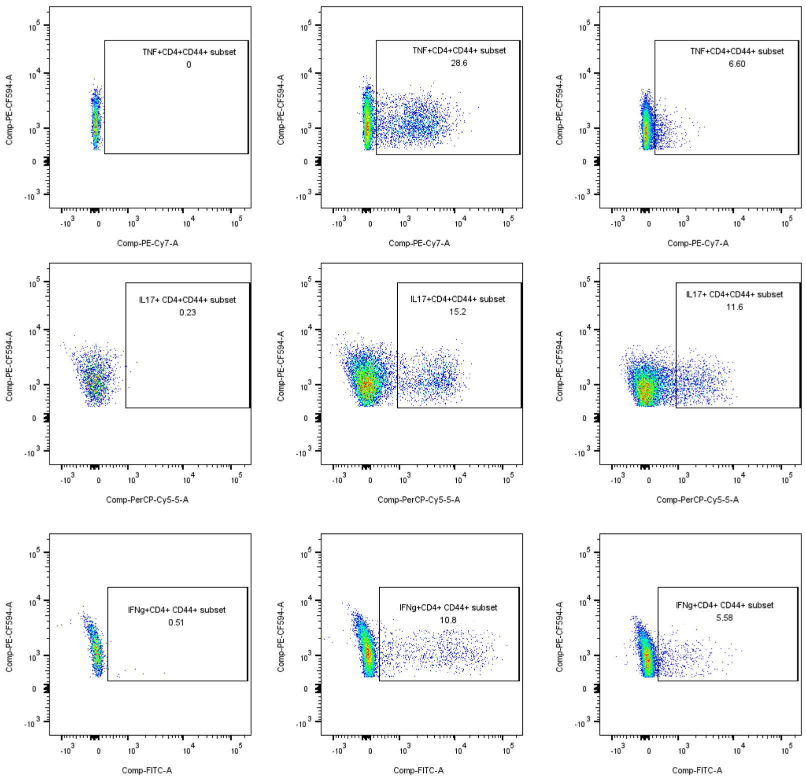

Supplementary Figure 4: Gating strategy for mucosal CD4+ T cell cytokine response post-vaccination. Mice were either vaccinated as described in Figure 3, Figure 4, Supplementary Figure 2 or Supplementary Figure 3 legends. Lung cells were isolated two weeks after the booster dose and restimulated in vitro with H56 antigen or left unstimulated (negative control). PMA

stimulation was included as positive control. Cells were stained with fluorescently labeled antibodies and analyzed by flow cytometry to assess cytokine production by $CD4^+CD44^+$ T cells. Representative gating strategy employed (A), and control plots from a single mouse used to define cytokine-positive gates based on unstimulated (negative) and PMA-stimulated (positive) conditions (B) are shown.
